# Early Postoperative Effects of Mechanical Versus Manual Femoral Canal Opening During Proximal Femoral Nail Antirotation Fixation of Pertrochanteric Femoral Fractures

**DOI:** 10.3390/medicina62071407

**Published:** 2026-07-20

**Authors:** Luka Roguljic, Veridijana Sunjic Roguljic, Bozen Pivalica, Marko Furlan, Ivana Banic, Matea Vidovic, Daniela Supe-Domic, Vedran Kovacic

**Affiliations:** 1 Surgery Department, Orthopaedics and Traumatology Division, University Hospital of Split, 21000 Split, Croatia; 2 Surgery Department, Plastic, Reconstructive and Aesthetic Surgery with Burn Care Division, University Hospital of Split, 21000 Split, Croatia; 3 School of Medicine, University of Split, 21000 Split, Croatia; 4 Division of Medical Laboratory Diagnostic, University Hospital of Split, 21000 Split, Croatia; 5 Internal Medicine Department, Division of Intensive Medicine with Clinical Pharmacology and Toxicology, University Hospital of Split, 21000 Split, Croatia

**Keywords:** femoral fractures, fracture fixation, intramedullary, inflammation, blood coagulation, bone remodelling

## Abstract

*Background and Objectives:* The method of femoral canal opening during intramedullary fixation may influence the biological response to surgery. The aim of this study was to compare the early biological responses associated with mechanical canal opening and manual canal opening during Proximal Femoral Nail Antirotation (PFNA) fixation in patients with pertrochanteric femoral fractures, with a particular focus on inflammation, coagulation, organ dysfunction, and bone metabolism. *Materials and Methods:* This single-centre prospective randomised study consisted of 60 participants (50 women) with a mean age of 80.33 ± 11.13 years, who were randomly assigned to either manual femoral canal opening (*n* = 30) or mechanical canal opening (*n* = 30). Outcomes were assessed preoperatively and postoperatively during a 72 h follow-up period. This study was registered at ClinicalTrials.gov (ClinicalTrials.gov ID NCT07648719; registration date: 15 June 2026; study start: 1 March 2025). *Results:* Across all participants, surgery was associated with a significant decrease in haemoglobin levels, calcium, and estimated glomerular filtration rate, while fibrinogen, urea, creatinine, CRP (C-reactive protein), and NT-proBNP (N-terminal pro-B-type natriuretic peptide) significantly increased postoperatively. Patients in the mechanical canal opening group demonstrated significantly higher postoperative levels of leukocytes (*p* = 0.002), CRP (*p* = 0.001), NT-proBNP (*p* = 0.002), fibrinogen (*p* = 0.026), activated partial thromboplastin time (*p* = 0.035), urea (*p* = 0.001), and creatinine (*p* = 0.018), as well as greater reductions in haemoglobin (*p* < 0.001), compared with the manual canal opening group. Bone turnover markers also differed between groups: postoperative bone-specific alkaline phosphatase levels were significantly higher in the mechanical canal opening group (*p* = 0.001), whereas the increase in total procollagen type 1 N-terminal propeptide was more pronounced in the manual canal opening group (*p* = 0.045). *Conclusions:* The method of femoral canal opening during PFNA fixation significantly influences early postoperative systemic inflammation, coagulability, organ stress, and bone turnover dynamics. Manual opening of the femoral canal was associated with lower early systemic and bone stress biomarker responses compared with mechanical canal opening. Whether these differences translate into improved clinical outcomes, particularly in elderly patients with limited physiological reserve, remains to be determined.

## 1. Introduction

Nearly 70 years ago, the German Association for the Study of Osteosynthesis (Arbeitsgemeinschaft für Osteosynthesefragen, AO) established four fundamental principles for intramedullary osteosynthesis: anatomical reduction, stable fixation, preservation of blood supply, and early mobilisation of operated patients [1]. The basis of intramedullary osteosynthesis in fractures of long bones is the insertion of a nail made of various materials, usually steel or titanium, into the medullary canal of the bone undergoing osteosynthesis [2].

The Proximal Femoral Nail Antirotation (PFNA) is a system developed by the AO in 2004 [3]. This method of intramedullary osteosynthesis with a metallic nail provides optimal anchorage and stability even when the implant is inserted into osteoporotic bone [4]. Bone compaction, together with the additional anchorage provided by the blades of the PFNA, ensures excellent stability of the osteosynthesis, which is particularly important in osteoporotic bone. The increased stability resulting from bone compaction around the PFNA blades reduces rotation and collapse [5].

A particular advantage of this method is that rotational and angular stability is achieved using only a single osteosynthetic element [6]. This technique has been proven effective in fractures of the proximal femur. The flexible type of osteosynthesis facilitates insertion and avoids stress at the tip of the PFNA. A medial–lateral angle of 6° allows insertion at the tip of the greater trochanter.

Intramedullary opening of the femoral medullary canal for PFNA insertion has been shown to cause a prolonged reduction in bone blood supply. The degree of cortical perfusion impairment correlates with the extent of opening, with evidence indicating that more extensive opening leads to a greater reduction in cortical blood flow [7]. In addition, opening has been associated with clinically relevant perioperative blood loss, which may reach up to 400 mL [8]. The increase in intramedullary pressure during the procedure facilitates the extrusion of bone marrow fat into the systemic circulation [9]. Furthermore, studies have demonstrated substantial heat generation during intramedullary opening, which in severe cases may result in thermal bone injury associated with thermal necrosis and disruption of endosteal architecture [10]. Proximal femoral fractures induce a substantial systemic inflammatory response caused by both the initial traumatic injury and the subsequent surgical treatment. Fracture healing is closely linked to activation of the immune system, involving leukocyte recruitment, cytokine release, coagulation pathway activation, and changes in bone turnover. In elderly patients, who often have limited physiological reserve, excessive postoperative inflammation may contribute to organ dysfunction and delayed recovery. Recent studies have demonstrated that blood-derived inflammatory biomarkers, including the neutrophil-to-lymphocyte ratio (NLR), platelet-to-lymphocyte ratio (PLR), and systemic immune-inflammation index (SII), correlate with the magnitude of surgical trauma in patients undergoing treatment for hip fractures [11]. Furthermore, postoperative inflammatory responses have been shown to differ according to the type and invasiveness of the surgical procedure, suggesting that technical aspects of fracture fixation may influence the biological response to surgery.

Intramedullary fixation with a PFNA device requires opening of the femoral medullary canal. Although this step is routinely performed, different techniques may be used, including manual canal and mechanical canal opening. The extent to which these techniques affect postoperative systemic inflammation, coagulation pathways, organ stress, and bone metabolism remains unclear.

The bone canal for insertion of the PFNA in pertrochanteric femoral fractures can be opened in two ways: by power using electric canal opening with a device that rapidly opens the medullary canal [12], or, alternatively, the medullary bone canal can be opened by manual opening using an orthopaedic awl, without the use of electric power [13,14]. When the entry into the medullary canal is not mechanically opened, there is possibly less endosteal trauma, and blood supply is maximised through minimally injured endosteal circulation. On the contrary, by mechanically opening the canal, the blood supply is immediately disrupted; however, this, in theory, may stimulate revascularization and bone healing [15].

To date, the influence of different techniques for femoral canal opening during the insertion of intramedullary osteosynthesis using PFNA on systemic inflammatory parameters, coagulation activity, and bone metabolism markers has not been sufficiently studied.

In particular, it is unknown whether there are differences in the aforementioned inflammatory and metabolic parameters depending on the two fundamental methods of femoral canal opening for insertion of the PFNA in pertrochanteric femoral fractures: mechanical or manual canal opening.

The aim of this study was to evaluate the effects of two techniques of femoral canal opening for insertion of the PFNA in pertrochanteric femoral fractures in patients randomised to either mechanical or manual canal opening on inflammation, coagulation, and bone metabolic parameters. A reduction in inflammatory and coagulation responses, together with more favourable bone metabolic biomarker profiles, may be associated with biological processes relevant to postoperative recovery. However, this study did not evaluate clinical outcomes such as perioperative complications, fracture healing, or functional recovery; therefore, the clinical implications of these findings require confirmation in future prospective studies. To the best of our knowledge, no previous study has directly compared manual femoral canal opening and mechanical canal opening during PFNA fixation with respect to their effects on systemic inflammatory response, coagulation status, and bone turnover.

## 2. Materials and Methods

A single-centre randomised prospective follow-up study was conducted from 1 March 2025 to 30 November 2025 at the Department of Surgery, Division of Orthopaedics and Traumatology, University Hospital in Split, Croatia. The study was designed as a single-blind trial, as outcome evaluators were blinded. This study was designed as an investigator-initiated, single-centre randomised surgical trial and received approval from the Ethics Committee of the University Hospital in Split (ethical code 520-03/25-01/65; approval date: 27 February 2025) before patient enrolment. Although the study was conducted according to the approved protocol, prospective registration in the public clinical trial registry was inadvertently overlooked. Consequently, the trial was registered retrospectively after completion of patient enrolment and primary data collection to ensure transparency and public accessibility of the study information. This study was registered at ClinicalTrials.gov (ClinicalTrials.gov ID NCT07648719; registration date: 15 June 2026; study start: 1 March 2025).

The study was conducted in accordance with the principles of the Declaration of Helsinki and the Consolidated Standards of Reporting Trials (CONSORT) guidelines. Informed consent was obtained from all participants included in the study.

### 2.1. Study Population

The study population consisted of adult patients (aged >18 years) admitted to the Department of Surgery, Orthopaedics and Traumatology Division, University Hospital Centre Split, who, during the observation period, sustained a pertrochanteric femoral fracture following a fall and were scheduled for surgery within 3 days after injury. Only patients with isolated unilateral pertrochanteric femoral fractures classified as AO/OTA (Arbeitsgemeinschaft für Osteosynthesefragen/Orthopaedic Trauma Association) 31A2 were included. Lateral wall involvement was defined as a disrupted or incompetent lateral femoral wall, including fractures with a lateral wall thickness of ≤20.5 mm.

Exclusion criteria included patients with polytrauma (defined as an Injury Severity Score (ISS) ≥ 16 or the presence of at least two clinically significant injuries involving different body regions); bilateral hip fractures; concomitant fractures of other skeletal regions; fracture-dislocations; pathological or periprosthetic fractures; previous surgery on the affected hip; patients with disseminated malignant disease; acute organ failure at the time of admission (including acute decompensated heart failure requiring hospital treatment, acute kidney injury according to stage 3 KDIGO criteria, acute respiratory failure requiring ventilatory support, or acute hepatic failure (characterized by liver dysfunction with coagulopathy and hepatic encephalopathy)); patients on vitamin D and calcium supplementation; autoimmune inflammatory diseases (e.g., rheumatoid arthritis, systemic lupus erythematosus); and patients receiving corticosteroids, cytostatics, anticoagulations, bisphosphonates, anabolics, antiresorptive bone-active drugs or immunosuppressive medications that could influence the observed parameters.

### 2.2. Study Flow

**Figure 1:**
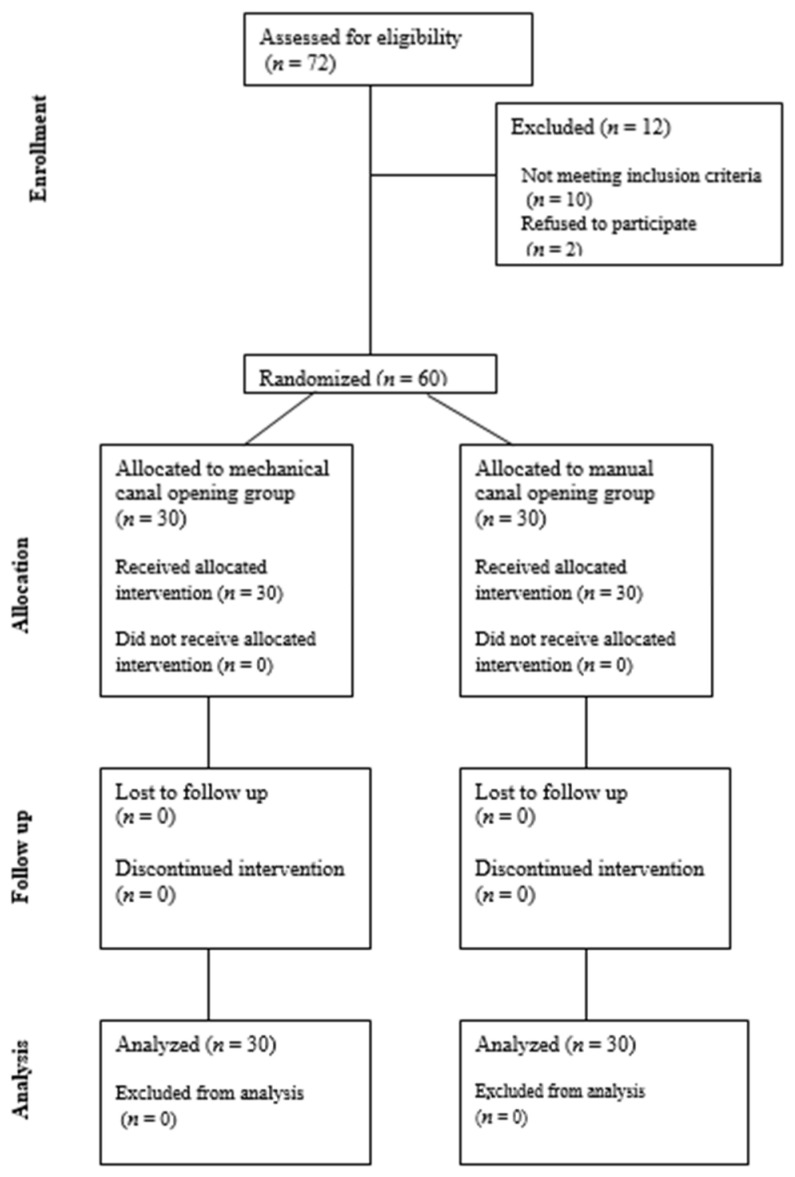
CONSORT flow diagram of the study.

During the study period, 72 patients were enrolled, and finally 60 patients were included in the analysis after applying exclusion criteria to 10 patients and accounting for 2 patients who declined participation. During the postoperative period, no patient withdrew or was lost to follow-up (Figure 1). The sample size was determined based on the expected primary outcome measure (C-reactive protein, CRP) derived from previous studies, using a significance level of α = 0.05 to detect a statistically significant difference and a study power of 90% (β = 0.1). Based on this power calculation, a minimum of 21 participants was estimated for each study group.

Patients were randomly assigned to either the mechanical canal opening group or the manual canal opening group. Randomization was computer-generated using random numbers in a 1:1 allocation ratio between the two interventions. Participants and trauma-orthopaedic surgeons were blinded to the allocation until entry into the operating room. Postoperative care and follow-up visits were identical in both groups. No patient experienced any treatment-related adverse effects.

### 2.3. Intervention Protocols

Each participant included in the study underwent surgery for a pertrochanteric femoral fracture. All participants were operated on according to the same protocol, using the same type of procedure on a traction table, with prior fracture reduction under fluoroscopic (X-ray) guidance. All fractures were reduced on a traction table under fluoroscopic guidance using a C-arm image intensifier (Siemens Healthineers, Erlangen, Germany) and fracture reduction was achieved intraoperatively. All patients had the same type of fracture (pertrochanteric femoral fracture), and intramedullary osteosynthesis was performed using a ⌀ 9.0 mm, 340 mm Proximal Femoral Nail Antirotation (PFNA) (Synthes GmbH, Oberdorf, Switzerland). The guidewire was introduced through the tip of the greater trochanter under fluoroscopic guidance, following the manufacturer’s recommended entry trajectory for PFNA insertion. Distal locking of the PFNA was achieved using one distal locking screw, with screw lengths ranging from 34 to 42 mm depending on patient anatomy.

All patients received subcutaneously the same weight-adjusted dose of low-molecular-weight heparin (dalteparin), as a single preoperative dose. Intraoperatively, all patients received a single dose of antibiotic prophylaxis (ceftriaxone 2 g intravenously), as well as postoperative analgesia with tramadol (20 mg intravenously) and a proton pump inhibitor, pantoprazole (40 mg intravenously).

In the first group, the medullary canal was opened under fluoroscopic guidance using a cannulated curved awl (14.0/3.2 mm). In the second group, the medullary canal was opened under fluoroscopic guidance using a battery-powered surgical opening equipped with a cannulated drill bit (13.0 mm diameter, 290 mm length) (Synthes GmbH, Oberdorf, Switzerland). No significant differences in operative time were observed between the groups. The mean duration of surgery was 20.37 ± 1.94 in the manual opening group and 20.33 ± 2.09 min in the mechanical opening group (*p* = 0.949). The mean duration of fluoroscopy was 0.56 ± 0.23 in the manual opening group and 0.56 ± 0.25 min in the mechanical opening group (*p* = 0.943).

During surgery, no drain was placed; therefore, blood loss via drainage could not be measured or followed, and was assessed only by blood haemoglobin concentration. In addition, no major intraoperative or early postoperative adverse events were recorded in either group that would have influenced the primary outcomes.

All patients were mobilised at the same time, on the second postoperative day. During hospitalisation, physical therapy and assisted walking with a physiotherapist were applied.

### 2.4. Estimation of Outcomes

This study had a prospective design. Outcomes were assessed preoperatively and during the follow-up period to 72 h after surgery. The 72 h follow-up period was chosen to capture the early postoperative biological response, as inflammatory markers, particularly CRP, typically peak within 48–72 h after surgery, while significant changes in coagulation and bone metabolism markers also occur during this interval. Bone turnover markers are primarily used to assess bone formation and remodelling processes over longer periods, typically several weeks after injury. In the present study, their assessment at 72 h postoperatively was not intended to evaluate fracture healing, but rather to explore early systemic metabolic responses to surgical trauma. The primary outcome of this study was the change in CRP levels between the study groups, and this parameter was used for the sample size calculation. Secondary outcomes included other inflammatory biomarkers, coagulation parameters, renal function markers, cardiac biomarkers, and bone turnover markers. During follow-up, all patients underwent laboratory testing. Blood samples were collected 2 h preoperatively and 72 h postoperatively in order to determine blood concentrations of leukocytes, erythrocytes, platelets (×10^9^/L), haemoglobin (g/L), D-dimers (IU/L), prothrombin time, international normalised ratio (INR), activated partial thromboplastin time (aPTT) (s), aPTT ratio, fibrinogen (g/L), creatinine (μmol/L), N-terminal pro-B-type natriuretic peptide (NT-proBNP) (pg/mL), C-reactive protein (CRP) (mg/L), glucose, urea, sodium, potassium, chloride, calcium, and phosphorus (mmol/L), as well as bone metabolism markers: total procollagen type 1 N-terminal propeptide (tP1NP) (µg/L), N-min osteocalcin (N-mOST) (µg/L), C-terminal telopeptide of type I collagen (β-CTX) (ng/mL), and bone-specific alkaline phosphatase (BAP) (µg/L).

We compared differences in blood parameters between the two groups at baseline, as well as differences after 72 h. In addition, we analysed the dynamics (baseline vs. 72 h) of outcome measures between the two study groups.

### 2.5. Statistical Analysis

Descriptive statistical analysis was performed, and results were presented as mean ± standard deviation. The normality of data distribution was assessed using the Shapiro–Wilk test. Differences in mean values of outcome measures between the two groups were analysed using the unpaired and paired two-tailed Student’s *t*-test. Correlations between variables were assessed using Pearson’s correlation coefficient. Given the relatively large number of biomarkers and correlations assessed and the exploratory nature of the study, no formal adjustment for multiple comparisons was performed. Therefore, the results should be interpreted as hypothesis-generating. Statistical analyses were performed using SPSS software for Windows (IBM SPSS Statistics for Windows, version 26.0; IBM Corp., Armonk, NY, USA). A *p*-value of <0.05 was considered statistically significant.

## 3. Results

A total of 60 participants were included in the study, comprising 50 women (83.3%) and 10 (16.7%) men. The mean age of the participants was 80.33 ± 11.13 years (range 44–97). After randomization, thirty patients underwent PFNA internal fixation following manual opening of the femoral canal, whereas thirty patients underwent PFNA internal fixation following mechanical opening of the femoral canal. There were no significant differences in patient age between the groups, and the male-to-female ratio was comparable in both groups.

Right-sided surgery was performed in 33 patients, while left-sided surgery was performed in 27 patients.

Outcome measures for all participants are presented in Table 1. There were differences between male (*n*= 10) and female (*n* = 50) participants in five outcomes: preoperative prothrombin time (ratio) (1.07 ± 0.10 vs. 0.96 ± 0.13, *p* = 0.015), preoperative fibrinogen (g/L) (3.24 ± 0.64 vs. 4.07 ± 1.21, *p* = 0.041), postoperative creatinine (µmol/L) (126.30 ± 69.19 vs. 95.06 ± 26.79, *p* = 0.017), postoperative phosphate (mmol/L) (1.12 ± 0.27 vs. 0.95 ± 0.14, *p* = 0.006) and postoperative BAP (µg/L) (15.20 ± 4.44 vs. 19.48 ± 6.32, *p* = 0.047) (Student’s *t*-test, two-tailed).

Differences in all outcome measures between the manual canal opening technique and the mechanical canal opening technique are presented in Table 2. As shown in Table 2a, comparison of the preoperative outcome parameters demonstrated no statistically significant differences between the manual and mechanical canal opening groups for the majority of baseline variables. Significant differences were observed only for preoperative leukocyte count, NT-proBNP, and serum potassium concentration, whereas all other inflammatory, coagulation, renal, and bone turnover markers were comparable between the groups. Although most baseline characteristics were comparable, these imbalances may have influenced subsequent between-group comparisons, particularly for parameters related to systemic inflammation and cardiometabolic stress. However, no covariate-adjusted analyses were performed, and all results are based on unadjusted comparisons, so the postoperative differences should be interpreted with caution.

Table 2b summarises the changes (delta values) in laboratory parameters from the preoperative assessment to the postoperative measurement in the two study groups. The primary outcome, CRP, demonstrated a significantly smaller postoperative increase in the manual canal opening group than in the mechanical canal opening group (36.51 ± 26.40 vs. 62.01 ± 31.12 mg/L, *p* = 0.001; 95% CI: −40.42 to −10.59).

Compared with the manual canal opening group, the mechanical canal opening group also showed significantly greater changes in leukocyte count, haemoglobin, D-dimer, urea, creatinine, NT-proBNP, and bone-specific alkaline phosphatase (BAP) (all *p* < 0.05).

**Figure 2:**
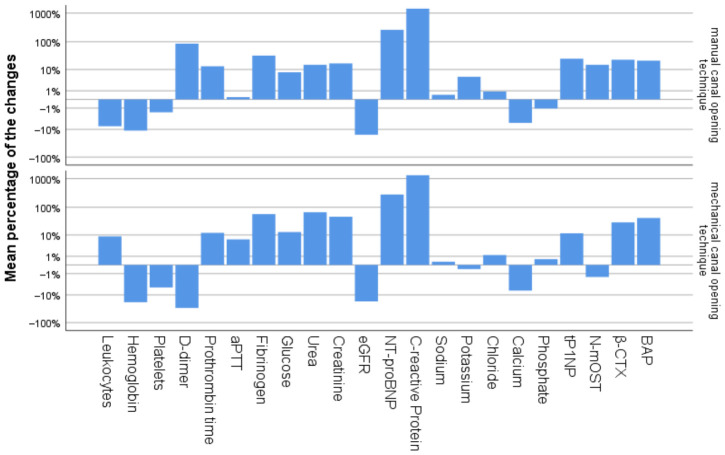
Differences in percentage changes (from baseline) of outcome measures between participants undergoing manual and mechanical canal opening techniques; data are depicted as mean percentage of the changes on a logarithmic scale (log10). The (**upper**) image represents the pattern of changes for manual canal opening bones, while the (**lower**) image represents the pattern of changes for mechanical canal opening bones. (aPTT: Activated partial thromboplastin time; eGFR: estimated glomerular filtration rate; tP1NP: total procollagen type 1 N-terminal propeptide; N-mOST: N-mid osteocalcin; β-CTX: beta-C-terminal telopeptide of type I collagen (B-CrossLaps); BAP: bone-specific alkaline phosphatase).

Table 3 and Figure 2 present the differences in the percentage change in outcome measures between the manual and mechanical canal opening techniques. Figure 2 depicts the pattern of changes from baseline values in both groups. Compared with the manual canal opening group, the mechanical canal opening group demonstrated significantly greater percentage changes in leukocyte count, haemoglobin, fibrinogen, urea, creatinine, and bone-specific alkaline phosphatase (BAP) (all *p* < 0.05). No significant between-group differences were observed for the CRP when expressed as a percentage change from baseline.

Differences in all outcome measures before and after surgery are presented in Table 4, Table 5 and Table 6 to provide a descriptive overview of the perioperative biological response within groups. When all participants were analysed together, significant postoperative changes were observed in several laboratory parameters compared with preoperative values. These included decreases in haemoglobin, estimated glomerular filtration rate (eGFR), calcium, and D-dimer, together with increases in prothrombin time, fibrinogen, urea, creatinine, NT-proBNP, C-reactive protein (CRP), tP1NP, β-CTX, and bone-specific alkaline phosphatase (BAP) (all *p* < 0.05). Within the manual canal opening group, significant postoperative changes were observed in haemoglobin, leukocyte count, prothrombin time, fibrinogen, creatinine, eGFR, NT-proBNP, CRP, calcium, tP1NP, β-CTX, and BAP (all *p* < 0.05). Within the mechanical canal opening group, significant postoperative changes were observed in haemoglobin, D-dimer, prothrombin time, fibrinogen, glucose, urea, creatinine, eGFR, NT-proBNP, CRP, calcium, β-CTX, and BAP (all *p* < 0.05).

Exploratory correlations between changes in bone turnover markers and changes in other outcome measures in participants with manual and mechanical canal opening groups are presented in Table 7 and Table 8. In the manual canal opening group, tP1NP was significantly correlated with platelet count and urea, N-mOST with D-dimer, fibrinogen, and phosphate, β-CTX with renal function parameters (urea, creatinine, and eGFR) and phosphate, and BAP with NT-proBNP and calcium. In the mechanical canal opening group, tP1NP was significantly associated with D-dimer, activated partial thromboplastin time (aPTT), and NT-proBNP; N-mOST with eGFR; β-CTX with calcium; and BAP with D-dimer, urea, and NT-proBNP. These correlation analyses revealed distinct patterns of association between bone turnover markers and perioperative laboratory parameters in the two treatment groups.

## 4. Discussion

This randomised prospective controlled study compared two different techniques of femoral canal opening—manual versus mechanical canal opening—during intramedullary fixation of pertrochanteric femoral fractures using the Proximal Femoral Nail Antirotation (PFNA). The main finding of this study is that the method of canal entry is associated with significant differences in systemic inflammatory response, coagulation dynamics, renal and cardiac stress markers, and bone turnover parameters within the first 72 postoperative hours. To the best of our knowledge, this is the first study to directly compare these two femoral canal opening techniques with a comprehensive analysis of their effects in the early postoperative period.

### 4.1. Systemic Inflammatory Response

A key observation of the study was a significantly higher systemic inflammatory response in the mechanical canal opening group, demonstrated by increased postoperative C-reactive protein (CRP) levels compared with the manual canal opening group. These findings suggest that mechanical canal opening may be associated with a more pronounced early postoperative inflammatory response compared with manual canal opening. This is consistent with the concept of “surgical trauma burden,” in which intramedullary manipulation increases the release of damage-associated molecular patterns, thereby activating innate immune pathways and cytokine cascades.

Postoperative serum biomarkers have increasingly been investigated as objective indicators of surgical tissue trauma and the magnitude of the biological response to different operative techniques. Previous studies have shown that intramedullary canal opening can significantly increase systemic inflammatory mediators, including interleukin-6 (IL-6) and CRP [16].

A recent systematic review and meta-analysis of randomised controlled trials comparing minimally invasive and conventional approaches in total hip arthroplasty demonstrated that postoperative biomarkers, including C-reactive protein, creatine kinase, and haemoglobin changes, can reflect differences in perioperative tissue injury and surgical stress between techniques [17]. The authors concluded that serum biomarkers may provide objective information regarding the extent of perioperative tissue injury and systemic response associated with surgical invasiveness. However, these biomarkers should be interpreted as surrogate indicators of tissue response rather than direct measures of clinical benefit. Although total hip arthroplasty and intramedullary fixation of pertrochanteric fractures represent different surgical procedures, the underlying concept that biochemical responses can reflect variations in surgical trauma is relevant to the present study. Similarly, in the present study, differences in postoperative inflammatory and other laboratory markers may reflect variations in the early biological response to femoral canal preparation, while their relationship with clinically meaningful outcomes remains uncertain.

Comparable findings have been described in intramedullary nailing of long-bone fractures, where the extent of canal opening correlates with systemic inflammatory activation [18]. Giannoudis et al. [19] demonstrated that extensive medullary instrumentation contributes to post-traumatic immunological activation and may aggravate the “second hit” phenomenon in trauma patients, although no difference was observed in the release of endothelial adhesion molecule markers, and no significant differences were found between groups treated with reamed and unreamed nailing. Marino et al. [20] demonstrated that PFNA fixation in pertrochanteric femoral fractures did not lead to a significant increase in serum levels of the inflammatory cytokines TNF-α and IL-6; however, CRP showed a significant rise on the first postoperative day, consistent with our findings.

Pape et al. [21], in a prospective study evaluating inflammatory cytokine release after orthopaedic trauma surgery during intramedullary fixation of femoral and pelvic fractures, demonstrated significantly increased perioperative IL-6 and IL-8 concentrations compared with patients treated for spinal fractures. The authors reported that the inflammatory response correlated with the degree of surgical invasiveness and blood loss. In particular, procedures involving intramedullary instrumentation induced the highest cytokine elevations, supporting the concept that manipulation of the medullary canal contributes substantially to systemic immune activation.

Although our study did not directly measure IL-6 or IL-8 concentrations, CRP and leukocyte counts represent downstream markers of cytokine-mediated systemic inflammation and likely reflect similar biological pathways. The findings of our study therefore support the hypothesis that even relatively small differences in intramedullary preparation techniques may influence the magnitude of early postoperative inflammatory biomarker responses.

The present results may be particularly relevant in elderly patients with hip fractures, who are highly susceptible to postoperative inflammatory dysregulation due to reduced physiological reserve and multiple comorbidities. However, whether modulation of these early biomarker responses influences clinically relevant outcomes remains unknown. Elevated postoperative inflammatory markers have been associated with delirium, impaired mobilisation, prolonged hospitalisation, delayed fracture healing, and increased mortality in geriatric trauma populations [22]. The AO principles emphasise biological preservation, and our findings suggest that differences in the invasiveness of femoral canal preparation may influence early postoperative inflammatory responses [23]. However, whether these biomarker differences reflect differences in local bone viability or translate into improved clinical outcomes remains uncertain.

### 4.2. Coagulation and Thrombo-Inflammatory Interaction

Both techniques were associated with significant postoperative changes in coagulation parameters, reflecting the expected hypercoagulable state after major orthopaedic surgery. Intramedullary manipulation is known to cause transient embolization of medullary contents and activation of coagulation pathways through tissue factor release and endothelial injury [24]. Opening of the femoral canal can also increase intramedullary pressure and fat embolization, which may amplify systemic coagulation and inflammatory responses [25]. Previous experimental and clinical studies have shown that intramedullary canal opening is associated with increased thrombin generation, platelet activation, and systemic inflammatory mediator release, supporting the concept that coagulation and inflammation are tightly interconnected biological processes during orthopaedic surgery [26].

The present study also demonstrated significant differences in coagulation-related parameters between the two femoral canal preparation techniques. Patients in the mechanical canal opening group showed more pronounced alterations in coagulation dynamics during the early postoperative period, suggesting that more extensive intramedullary instrumentation may be associated with differences in early haemostatic responses. Similarly, Albert et al. [27] analysed acute changes in coagulation, fibrinolysis, and inflammatory responses following intramedullary nailing of long bones, as well as their potential impact on clinical outcomes. The authors demonstrated that intramedullary instrumentation leads to significant activation of coagulation and inflammatory pathways, including increased inflammatory cytokines and markers of endothelial activation. The findings of this study are consistent with our results, showing that mechanical femoral canal opening during PFNA implantation is associated with a more pronounced inflammatory and coagulation response. Although the cited study evaluated intramedullary nailing in general rather than the specific canal entry technique, both studies suggest that the extent of intramedullary manipulation plays an important role in activating thrombo-inflammatory mechanisms.

The interaction between coagulation and inflammation is particularly relevant in elderly patients with pertrochanteric fractures, who frequently present with endothelial dysfunction, frailty, and pre-existing prothrombotic conditions. In such patients, exaggerated thrombo-inflammatory activation may contribute to postoperative complications, including venous thromboembolism, pulmonary dysfunction, microvascular impairment, and delayed tissue recovery. Robinson et al. [28] demonstrated that open intramedullary nailing induces significant activation of coagulation and fibrinolytic pathways, accompanied by marrow embolization and transient cardiopulmonary changes, including an increased risk of postoperative respiratory complications and transient right-sided cardiac strain during femoral canal instrumentation. Our results support the hypothesis that differences in femoral canal preparation technique may influence early thrombo-inflammatory biomarker responses. The clinical relevance of these differences remains uncertain and requires further investigation.

The greater postoperative decrease in haemoglobin observed in the mechanical canal opening group may suggest greater perioperative blood loss, possibly related to more extensive endosteal vascular disruption associated with this technique. However, as intraoperative blood loss was not directly measured, these findings should be interpreted with caution.

Mechanical canal opening generates more extensive trabecular and cortical microdamage compared with manual opening, which may enhance local bleeding. These findings are consistent with previous reports suggesting that more invasive canal preparation techniques are associated with greater surgical trauma and perioperative blood loss, as described in studies comparing canal opening with less invasive intramedullary entry methods [29,30].

### 4.3. Renal and Cardiac Stress

Patients in the mechanical canal opening group demonstrated more pronounced deterioration of renal function together with higher postoperative NT-proBNP levels, indicating a greater degree of systemic physiological burden following surgery. Despite the baseline imbalance in NT-proBNP between groups, a significant difference in its change over time was observed; nevertheless, all analyses were unadjusted, and the results should therefore be interpreted with caution in this context. As no covariate-adjusted analyses were performed, the independent contribution of the canal opening technique to these biomarker differences cannot be definitively established.

These findings may reflect differences in the early postoperative biological response following the two canal opening techniques. Several mechanisms, including inflammatory activation, coagulation response, marrow embolization, and transient hemodynamic stress induced by more extensive intramedullary manipulation, may contribute to the observed biomarker differences; however, these mechanisms were not directly assessed in the present study.

The relationship between orthopaedic surgery and postoperative organ dysfunction has been increasingly recognised in recent years. Intramedullary instrumentation can induce embolization of medullary contents, endothelial activation, cytokine release, and microvascular dysfunction, potentially contributing to transient impairment of renal perfusion and cardiac strain. Robinson et al. [28] demonstrated that open intramedullary nailing is associated with significant coagulative activation, embolic phenomena, pulmonary vascular changes, and right-sided cardiac strain.

Elevated NT-proBNP likely reflects perioperative cardiac strain in this high-risk elderly population rather than primary cardiac pathology; however, the increase was significantly greater in the mechanical canal opening group. The observed increase in NT-proBNP in the mechanical canal opening group may reflect a transient perioperative cardiac stress response occurring in the early postoperative period. This finding may be associated with perioperative physiological changes such as alterations in haemodynamics and systemic inflammatory response during surgery; however, the exact underlying mechanisms cannot be determined from the present data. NT-proBNP has been shown to correlate with postoperative cardiac complications and adverse outcomes in elderly patients undergoing hip fracture surgery. Cuthbertson et al. [31] reported that elevated perioperative natriuretic peptide levels are associated with increased cardiovascular risk and postoperative morbidity in non-cardiac surgery, while Oscarsson et al. [32] demonstrated that NT-proBNP is an independent predictor of postoperative cardiac events in orthopaedic and other high-risk surgical patients. Although NT-proBNP levels increased postoperatively, these changes should be interpreted cautiously in the absence of echocardiographic or cardiac troponin data. Accordingly, NT-proBNP in this study is considered a non-specific marker of perioperative cardiometabolic and systemic stress rather than a direct indicator of myocardial dysfunction. The observed elevation is likely multifactorial and may also be influenced by systemic inflammatory and hemodynamic responses to surgical trauma.

Both groups demonstrated postoperative deterioration in renal function markers (increased creatinine and reduced eGFR), which is expected in elderly patients after trauma and surgery. However, these changes were significantly more pronounced in the mechanical canal opening group. Postoperative renal dysfunction after major orthopaedic procedures, particularly hip fracture surgery in elderly patients, is increasingly recognised as a frequent and clinically relevant complication [33,34]. Renal dysfunction after major orthopaedic procedures has likewise been linked to systemic inflammatory activation, oxidative stress, embolic microcirculatory impairment, and perioperative hemodynamic instability [35]. Studies have demonstrated that pre-existing renal impairment, hypoalbuminemia, and advanced age are strong predictors of postoperative renal failure, highlighting the vulnerability of this cohort to even modest perioperative insults [36]. Even subtle postoperative increases in creatinine may be clinically relevant in elderly hip fracture patients. The more pronounced alterations observed in the mechanical canal opening group may indicate a higher degree of renal susceptibility to thrombo-inflammatory activation or possible embolic phenomena, supporting the hypothesis that even minor variations in surgical technique can influence early postoperative renal function in frail orthopaedic patients. Although the observed differences may indicate a more favourable early biological response, the present study was not designed to evaluate clinical outcomes. Therefore, any potential clinical implications of these findings should be interpreted with caution and require confirmation in future studies specifically designed to assess clinical outcomes.

### 4.4. Bone Metabolism Biomarkers

An additional important aspect of our study was the evaluation of bone turnover biomarkers, which provided insight into the early biological response of bone tissue following PFNA implantation. Bone remodelling after fracture fixation is a highly coordinated process involving both osteoblastic bone formation and osteoclastic resorption. In the present study, differences observed between the mechanical and manual canal opening groups in bone metabolism markers suggest that the method of femoral canal opening may influence early postoperative bone metabolism and remodelling dynamics.

The significant differences in tP1NP and BAP observed between the study groups may reflect differences in early bone turnover responses following the two surgical techniques. tP1NP is considered a sensitive marker of early type I collagen synthesis and initial osteoblastic bone formation, whereas BAP reflects later osteoblastic activity associated with matrix maturation and mineralization [37]. Elevation of BAP may indicate increased reparative bone turnover following more extensive cortical and endosteal disruption [38]. Given the short 72 h follow-up period, changes in bone turnover markers (tP1NP and BAP) should be interpreted cautiously. At this early stage, they primarily reflect an acute postoperative response to surgical stress rather than true bone remodelling or fracture healing. Longer follow-up is required to determine their clinical relevance.

The greater postoperative increase in tP1NP observed in the manual opening group may reflect better preservation of endosteal vascularization and reduced thermal or mechanical bone injury, potentially enabling a more physiological early reparative response with enhanced collagen synthesis. In contrast, the higher BAP levels in the mechanical canal opening group may represent a compensatory osteoblastic response to more extensive canal preparation, while the relatively lower tP1NP levels may reflect differences in the timing and phase of early bone turnover rather than a true suppression of collagen synthesis.

Conversely, the different osteometabolic profiles observed between groups may reflect variations in early bone turnover responses following surgical intervention. However, given the short follow-up period and the absence of radiological or functional healing outcomes, these biomarker changes cannot be interpreted as evidence of improved fracture healing or superior biological performance of either technique. Stewart et al. [39] reported that tP1NP and CTX (C-terminal telopeptide of type I collagen) concentrations after intramedullary fixation of tibial and femoral fractures were associated with radiographic healing progression, supporting the concept that bone turnover markers may reflect the biological processes underlying fracture repair.

Similar findings have been reported in studies evaluating fracture healing and intramedullary fixation, where increased surgical trauma and local bone remodelling have been associated with higher postoperative BAP levels and enhanced osteoblastic activity during the early reparative phase [40].

Conversely, the different biomarker profile observed in the manual canal opening group may reflect differences in the early biological response to surgical intervention. Experimental studies have shown that changes in intramedullary pressure and endosteal vascular disruption are key regulators of osteogenesis and fracture healing biology [41]. These findings suggest that different femoral canal opening techniques may modulate distinct phases of postoperative bone remodelling and osteoblastic activity.

The correlation analysis revealed different patterns of interaction between changes in bone turnover markers and systemic responses in the two study groups. In the manual canal opening group, changes in bone formation and resorption markers showed more consistent associations with coagulation, renal function, and fibrinolytic activity.

In the mechanical canal opening group, the correlation pattern was more pronounced and more consistent, with stronger associations between changes in bone turnover markers and markers of coagulation and cardiovascular stress. A particularly notable finding was the strong significant positive correlation between changes in tP1NP and aPTT, as well as between changes in tP1NP and D-dimers. Furthermore, changes in BAP showed a significant association with NT-proBNP, potentially reflecting a link between more intensive bone remodelling and cardiovascular stress. In addition, correlations with renal parameters were observed, further suggesting that mechanical canal opening may be associated with a different pattern of associations between bone turnover markers and systemic biomarkers. Taken together, these findings suggest that different femoral canal preparation techniques may be associated with distinct patterns of early postoperative biomarker responses.

### 4.5. Limitations

This study has several limitations. First, it is a single-centre study with a relatively small sample size and reduced statistical power, which may limit the generalizability of the findings. Furthermore, although randomization was performed, the possibility of residual baseline imbalance between groups cannot be completely excluded. Despite possible baseline imbalances between groups, no covariate-adjusted analyses were performed, and all results are based on unadjusted comparisons. Second, follow-up was limited to 72 h, capturing only early postoperative biological responses without assessing long-term outcomes such as fracture healing time or implant failure. Third, potential confounders such as individual variations in bone quality, comorbidities, and perioperative hemodynamics may have influenced biomarker levels. The distribution of fracture subtypes was not fully stratified in the analysis, which may represent a potential source of heterogeneity between groups. Fourth, the study did not include direct intraoperative measurements such as blood loss, intramedullary pressure, embolic load, or inflammatory cytokines (e.g., interleukin-6), which limits the mechanistic interpretation of the observed biochemical changes. Finally, the present study focuses on early postoperative biological and biochemical responses rather than clinically definitive outcomes such as thromboembolic events, rehospitalization, or mortality. Therefore, the observed differences in inflammatory, coagulation, and bone metabolism markers should be interpreted as early biological responses and surrogate endpoints, rather than indicators of direct clinical benefit. The clinical significance of these findings remains uncertain and requires further investigation in studies with long-term follow-up and clinically relevant outcome measures.

## 5. Conclusions

These findings suggest that even relatively small technical modifications during intramedullary nail insertion may influence the early systemic biological responses. Compared with manual canal opening, mechanical opening of the femoral canal was associated with a more pronounced systemic inflammatory and coagulation response, greater postoperative haemoglobin reduction (suggesting a possible increase in postoperative bleeding), increased renal and cardiac stress, and greater activation of bone turnover in the early postoperative period. These findings indicate differences in biological responses following the two techniques; however, laboratory biomarkers represent surrogate endpoints and should not be interpreted as evidence of a superior surgical technique or improved clinical outcomes. This may be particularly relevant in elderly patients with limited physiological reserve. Further multicentre studies incorporating comprehensive clinical and functional outcome measures are warranted to confirm and validate these findings.

## Figures and Tables

**Table 1:** Outcome measures for all participants (pre- and postoperative values).

Parameter	Preoperative (Mean ± SD)	Postoperative (Mean ± SD)	Minimum–Maximum
Leukocytes (×10^9^/L)	11.62 ± 3.34	11.23 ± 3.29	5.90–18.10
Haemoglobin (g/L)	120.40 ± 18.52	101.77 ± 14.44	80–168
Platelets (×10^9^/L)	214.77 ± 60.32	206.27 ± 70.61	40–446
D-dimer (µg/L)	12.58 ± 10.92	6.72 ± 6.86	1.25–35.20
Prothrombin time (ratio)	0.98 ± 0.13	1.09 ± 0.14	0.64–1.45
aPTT (ratio)	23.88 ± 2.72	24.55 ± 3.27	17.60–33.30
Fibrinogen (g/L)	3.93 ± 1.17	5.31 ± 1.36	1.60–9.00
Glucose (mmol/L)	7.57 ± 2.22	7.81 ± 2.45	2.80–18.30
Urea (mmol/L)	7.77 ± 3.72	9.77 ± 3.87	1.90–22.10
Creatinine (µmol/L)	78.02 ± 23.67	100.27 ± 38.26	41–316
eGFR (mL/min/1.73 m^2^)	68.08 ± 24.03	56.39 ± 21.88	9.20–107.20
NT-proBNP (pg/mL)	655.40 ± 1053.96	1537.33 ± 1596.93	64–6124
C-reactive protein (mg/L)	21.10 ± 24.99	70.36 ± 35.94	0.60–168
Sodium (mmol/L)	138.58 ± 4.46	139.03 ± 4.15	127–148
Potassium (mmol/L)	4.20 ± 0.54	4.26 ± 0.48	2.50–5.60
Chloride (mmol/L)	101.77 ± 5.05	102.70 ± 4.69	88–113
Calcium (mmol/L)	2.26 ± 0.11	2.12 ± 0.19	1.16–2.80
Phosphate (mmol/L)	1.00 ± 0.21	0.98 ± 0.18	0.44–1.57
tP1NP (µg/L)	52.56 ± 20.48	59.18 ± 26.75	14.40–141
N-mOST (µg/L)	25.41 ± 24.64	23.31 ± 15.89	4.50–193
β-CTX (ng/mL)	0.62 ± 0.28	0.72 ± 0.27	0.11–1.51
BAP (µg/L)	14.56 ± 5.14	18.76 ± 6.23	5.50–34.80

Legend: aPTT: Activated partial thromboplastin time; eGFR: estimated glomerular filtration rate; NT proBNP: N-terminal pro-B-type natriuretic peptide; tP1NP: total procollagen type 1 N-terminal propeptide; N-mOST: N-mid osteocalcin; β-CTX: beta-C-terminal telopeptide of type I collagen (B-CrossLaps); BAP: bone-specific alkaline phosphatase.

**Table 2:** Differences in baseline, postoperative and delta outcome measures (delta values from beginning to postprocedural) between manual canal opening technique (*n* = 30) and mechanical canal opening technique (*n*= 30) (unpaired Student’s *t*-test, two-tailed).

	Manual Canal Opening Technique	Mechanical Canal Opening Technique		95% Confidence Interval of the Difference	
	Mean ± Std. Deviation	Mean ± Std. Deviation	*p*	Lower	Upper
**(a) Baseline and postoperative outcome measures**					
Age (years)	82.03 ± 10.97	78.63 ± 11.21	0.240	−2.33	9.13
pre_Leukocytes (×10^9^/L)	10.72 ± 3.07	12.53± 3.40	**0.035**	−3.48	−0.13
post_Leukocytes (×10^9^/L)	9.33 ± 2.44	13.14 ± 2.93	**<0.001**	−5.21	−2.42
pre_Haemoglobin (g/L)	117.23 ± 15.41	123.57 ± 20.97	0.188	−15.84	3.18
post_Haemoglobin (g/L)	103.93 ± 14.06	99.60 ± 14.72	0.248	−3.10	11.77
pre_Platelets (×10^9^/L)	214.47 ± 71.59	215.07 ± 47.72	0.970	−32.04	30.84
post_Platelets (×10^9^/L)	211.37 ± 86.87	201.17 ± 50.43	0.580	−26.51	46.91
pre_D-dimer (µg/L)	11.56 ± 11.12	13.55 ± 10.81	0.489	−7.71	3.73
post_D-dimer (µg/L)	8.77 ± 8.77	4.67 ± 3.19	**0.019**	0.69	7.51
pre_Prothrombin time (ratio)	0.96 ± 0.13	1.00 ± 0.13	0.225	−0.11	0.03
post_Prothrombin time (ratio)	1.07 ± 0.13	1.11 ± 0.15	0.297	−0.11	0.03
pre_aPTT (ratio)	24.10 ± 2.90	23.66 ± 2.54	0.535	−0.97	1.85
post_aPTT (ratio)	24.01 ± 2.66	25.08 ± 3.76	0.211	−2.75	0.62
pre_Fibrinogen (g/L)	4.19 ± 1.27	3.67 ± 1.03	0.087	−0.08	1.12
post_Fibrinogen (g/L)	5.13 ± 1.20	5.48 ± 1.50	0.322	−1.05	0.35
pre_Glucose (mmol/L)	7.44 ± 2.54	7.70 ± 1.88	0.645	−1.42	0.89
post_Glucose (mmol/L)	7.01 ± 2.17	8.61 ± 2.48	**0.010**	−2.81	−0.39
pre_Urea (mmol/L)	8.01 ± 3.60	7.53 ± 3.87	0.621	−1.45	2.41
post_Urea (mmol/L)	8.78 ± 3.40	10.76 ± 4.10	**0.047**	−3.92	−0.03
pre_Creatinine (µmol/L)	79.50 ± 24.66	76.53 ± 22.96	0.631	−9.35	15.28
post_Creatinine (µmol/L)	93.87 ± 49.18	106.67 ± 21.80	0.198	−32.46	6.86
pre_eGFR (mL/min/1.73 m^2^)	67.26 ± 21.56	68.90 ± 26.62	0.793	−14.17	10.87
post_eGFR (mL/min/1.73 m^2^)	55.57 ± 20.25	57.21 ± 23.71	0.774	−13.04	9.76
pre_NT-proBNP (pg/mL)	385.23 ± 523.33	925.57± 1354.64	**0.046**	−1071.06	−9.60
post_NT-proBNP (pg/mL)	870.07 ± 750.10	2204.60± 1924.70	**0.001**	−2089.47	−579.60
pre_C-reactive protein (mg/L)	20.56 ± 23.20	21.65 ± 27.04	0.868	−14.11	11.94
post_C-reactive protein (mg/L)	57.07 ± 31.74	83.66 ± 35.42	**0.003**	−43.98	−9.21
pre_Sodium (mmol/L)	138.87 ± 3.73	138.30 ± 5.14	0.627	−1.75	2.89
post_Sodium (mmol/L)	139.43 ± 4.03	138.63 ± 4.30	0.460	−1.35	2.95
pre_Potassium (mmol/L)	4.06 ± 0.46	4.34 ± 0.57	**0.042**	−0.55	−0.01
post_Potassium (mmol/L)	4.26 ± 0.54	4.25 ± 0.41	0.979	−0.25	0.25
pre_Chloride (mmol/L)	102.10 ± 4.71	101.43 ± 5.42	0.613	−1.96	3.29
post_Chloride (mmol/L)	102.83 ± 4.73	102.57 ± 4.72	0.828	−2.18	2.71
pre_Calcium (mmol/L)	2.27 ± 0.11	2.25 ± 0.12	0.455	−0.04	0.08
post_Calcium (mmol/L)	2.14 ± 0.11	2.10 ± 0.25	0.341	−0.05	0.15
pre_Phosphate (mmol/L)	1.02 ± 0.18	0.98 ± 0.25	0.533	−0.08	0.15
post_Phosphate (mmol/L)	1.00 ± 0.18	0.95 ± 0.17	0.256	−0.04	0.14
pre_tP1NP (µg/L)	56.31 ± 22.45	48.81 ± 17.89	0.158	−2.99	17.99
post_tP1NP (µg/L)	67.38 ± 33.24	50.97 ± 14.56	**0.016**	3.15	29.67
pre_N-mOST (µg/L)	23.68 ± 13.04	27.15 ± 32.55	0.590	−16.28	9.35
post_N-mOST (µg/L)	26.14 ± 20.73	20.48 ± 8.21	0.170	−2.49	13.81
pre_β-CTX (ng/mL)	0.64 ± 0.30	0.61 ± 0.26	0.634	−0.11	0.18
post_β-CTX (ng/mL)	0.74 ± 0.28	0.71 ± 0.26	0.595	−0.10	0.18
pre_BAP (µg/L)	14.42 ± 5.65	14.69 ± 4.66	0.841	−2.95	2.41
post_BAP (µg/L)	17.25 ± 6.41	20.28 ± 5.75	0.059	−6.18	0.11
**(b) Changes in outcome measures (delta values from baseline to postoperative)**					
**Delta**					
Leukocytes (×10^9^/L)	−1.39 ± 2.77	0.61 ± 2.52	**0.005**	−3.37	−0.64
Haemoglobin (g/L)	−13.30 ± 10.00	−23.97 ± 12.16	**<0.001**	4.91	16.42
Platelets (×10^9^/L)	−3.10 ± 49.75	−13.90 ± 48.28	0.397	−14.54	36.14
D-dimer (µg/L)	−2.41 ± 13.17	−8.88 ± 11.71	**0.049**	0.03	12.91
Prothrombin time (ratio)	0.12 ± 0.14	0.11 ± 0.13	0.917	−0.07	0.07
aPTT (ratio)	−0.08 ± 1.86	1.42 ± 4.06	0.070	−3.13	0.13
Fibrinogen (g/L)	0.94 ± 1.72	1.81 ± 1.68	0.052	−1.75	0.01
Glucose (mmol/L)	−0.43 ± 3.36	0.90 ± 1.79	0.060	−2.72	0.06
Urea (mmol/L)	0.77 ± 2.61	3.23 ± 2.89	**0.001**	−3.88	−1.03
Creatinine (µmol/L)	14.37 ± 34.56	30.13 ± 20.16	**0.035**	−30.39	−1.14
eGFR (mL/min/1.73 m^2^)	−11.69± 12.24	−11.70 ± 9.81	0.998	−5.73	5.74
NT-proBNP (pg/mL)	484.83 ± 427.52	1279.03 ± 1406.93	**0.004**	−1331.59	−256.81
C-reactive protein (mg/L)	36.51 ± 26.40	62.01 ± 31.12	**0.001**	−40.42	−10.59
Sodium (mmol/L)	0.57 ± 3.52	0.33 ± 3.55	0.799	−1.59	2.06
Potassium (mmol/L)	0.19 ± 0.52	−0.09 ± 0.72	0.085	−0.04	0.61
Chloride (mmol/L)	0.73 ± 4.29	1.13 ± 3.61	0.697	−2.45	1.65
Calcium (mmol/L)	−0.13 ± 0.11	−0.15 ± 0.23	0.575	−0.07	0.12
Phosphate (mmol/L)	−0.01 ± 0.20	−0.06 ± 0.33	0.485	−0.09	0.19
tP1NP (µg/L)	11.07 ± 24.97	2.16 ± 13.30	0.090	−1.43	19.25
N-mOST (µg/L)	2.46 ± 17.48	−6.67 ± 32.15	0.177	−4.24	22.50
β-CTX (ng/mL)	0.10 ± 0.18	0.10 ± 0.23	0.965	−0.10	0.11
BAP (µg/L)	2.83 ± 3.27	5.59 ± 3.23	**0.002**	−4.44	−1.08

Legend: *p*: significance level; aPTT: Activated partial thromboplastin time; eGFR: estimated glomerular filtration rate; NT proBNP: N-terminal pro-B-type natriuretic peptide; tP1NP: total procollagen type 1 N-terminal propeptide; N-mOST: N-mid osteocalcin; β-CTX: beta-C-terminal telopeptide of type I collagen (B-CrossLaps); BAP: bone-specific alkaline phosphatase. Statistically significant values (*p* < 0.05) are shown in bold.

**Table 3:** Differences in percentage changes (from baseline values) of outcome measures between participants undergoing the manual canal opening technique (*n* = 30) and the mechanical canal opening technique (*n* = 30) (unpaired Student’s two-tailed *t*-test).

	Manual Canal Opening Technique	Mechanical Canal Opening Technique		95% Confidence Interval of the Difference	
Percentage of Delta (%)	Mean± Std. Deviation	Mean± Std. Deviation	*p*	Lower	Upper
Leukocytes	−8.40 ± 31.01	8.84 ± 24.20	**0.020**	−0.32	−0.03
Haemoglobin	−10.99 ± 8.42	−18.64 ± 8.31	**0.001**	0.03	0.12
Platelets	−1.81 ± 22.27	−5.08 ± 21.19	0.563	−0.08	0.14
D-dimer	85.98 ± 386.78	−30.31 ± 65.70	0.110	−0.27	2.60
Prothrombin time	13.42 ± 16.01	12.10 ± 14.26	0.737	−0.07	0.09
aPTT	0.06 ± 7.73	6.73 ± 16.61	0.051	−0.13	0.00
Fibrinogen	33.01 ± 47.45	57.44 ± 46.50	**0.049**	−0.49	0.00
Glucose	7.91 ± 84.19	12.94 ± 22.52	0.753	−0.37	0.27
Urea	15.76 ± 34.20	66.84 ± 85.60	**0.004**	−0.85	−0.17
Creatinine	16.56 ± 29.57	46.19 ± 34.53	**0.001**	−0.46	−0.13
eGFR	−16.03± 23.64	−17.45 ± 13.75	0.778	−0.09	0.11
NT-proBNP	261.87 ± 240.12	278.13 ± 363.50	0.839	−1.75	1.43
C-reactive protein	1380.31 ± 2487.78	1318.62 ± 1893.80	0.914	−10.81	12.04
Sodium	0.43 ± 2.52	0.29 ± 2.56	0.831	−0.01	0.01
Potassium	5.52 ± 13.90	−0.40 ± 16.28	0.135	−0.02	0.14
Chloride	0.80 ± 4.21	1.22 ± 3.60	0.685	−0.02	0.02
Calcium	−5.47 ± 4.70	−6.78 ± 10.12	0.524	−0.03	0.05
Phosphate	0.64 ± 20.27	0.59 ± 40.99	0.995	−0.17	0.17
tP1NP	24.46 ± 54.85	11.64 ± 30.96	0.269	−0.10	0.36
N-mOST	14.73 ± 88.81	−1.62 ± 32.79	0.348	−0.18	0.51
β-CTX	27.05 ± 40.05	29.16 ± 51.84	0.860	−0.26	0.22
BAP	21.27 ± 25.35	41.87 ± 30.60	**0.006**	−0.35	−0.06

Legend: *p*: significance level; aPTT: Activated partial thromboplastin time; eGFR: estimated glomerular filtration rate; NT proBNP: N-terminal pro-B-type natriuretic peptide; tP1NP: total procollagen type 1 N-terminal propeptide; N-mOST: N-mid osteocalcin; β-CTX: beta-C-terminal telopeptide of type I collagen (B-CrossLaps); BAP: bone-specific alkaline phosphatase. Statistically significant values (*p* < 0.05) are shown in bold.

**Table 4:** Differences in all outcome measures before and after surgery in all participants (data presented as mean ± standard deviation, paired Student’s *t*-test, two-tailed).

	Before	After	*p*	95% Confidence Interval of the Difference	
				Lower	Upper
Leukocytes (×10^9^/L)	11.62 ± 3.34	11.23 ± 3.29	0.287	−0.34	1.12
Haemoglobin (g/L)	120.40 ± 18.52	101.77 ± 14.44	**<0.001**	15.46	21.81
Platelets (×10^9^/L)	214.77 ± 60.32	206.27 ± 70.61	0.183	−4.13	21.13
D-dimer (µg/L)	12.58 ± 10.92	6.78 ± 6.90	**0.001**	2.45	9.14
Prothrombin time (ratio)	0.98 ± 0.13	1.09 ± 0.14	**<0.001**	−0.15	−0.08
aPTT (ratio)	23.88 ± 2.72	24.55 ± 3.27	0.113	−1.50	0.16
Fibrinogen (g/L)	3.93 ± 1.17	5.31 ± 1.36	**<0.001**	−1.83	−0.93
Glucose (mmol/L)	7.57 ± 2.22	7.81 ± 2.45	0.508	−0.95	0.47
Urea (mmol/L)	7.77 ± 3.72	9.77 ± 3.87	**<0.001**	−2.78	−1.23
Creatinine (µmol/L)	78.02 ± 23.67	100.27 ± 38.26	**<0.001**	−29.78	−14.72
eGFR (mL/min/1.73 m^2^)	68.08± 24.03	56.39 ± 21.88	**<0.001**	8.85	14.53
NT-proBNP (pg/mL)	655.40 ± 1053.96	1537.33 ± 1596.93	**<0.001**	−1167.63	−596.23
C-reactive protein (mg/L)	21.10 ± 24.99	70.36 ± 35.94	**<0.001**	−57.36	−41.16
Sodium (mmol/L)	138.58 ± 4.46	139.03 ± 4.15	0.324	−1.36	0.46
Potassium (mmol/L)	4.20 ± 0.54	4.26 ± 0.48	0.532	−0.22	0.11
Chloride (mmol/L)	101.77 ± 5.05	102.70 ± 4.69	0.071	−1.95	0.08
Calcium (mmol/L)	2.26 ± 0.11	2.12 ± 0.19	**<0.001**	0.09	0.19
Phosphate (mmol/L)	1.00 ± 0.22	0.98 ± 0.18	0.506	−0.04	0.08
tP1NP (µg/L)	52.56 ± 20.48	59.18 ± 26.75	**0.014**	−11.87	−1.36
N-mOST (µg/L)	25.41 ± 24.64	23.31 ± 15.89	0.535	−4.63	8.83
β-CTX (ng/mL)	0.62 ± 0.28	0.72 ± 0.27	**<0.001**	−0.15	−0.05
BAP (µg/L)	14.56 ± 5.14	18.76 ± 6.23	**<0.001**	−5.12	−3.30

Legend: *p*: significance level; aPTT: Activated partial thromboplastin time; eGFR: estimated glomerular filtration rate; NT proBNP: N-terminal pro-B-type natriuretic peptide; tP1NP: total procollagen type 1 N-terminal propeptide; N-mOST: N-mid osteocalcin; β-CTX: beta-C-terminal telopeptide of type I collagen (B-CrossLaps); BAP: bone-specific alkaline phosphatase. Statistically significant values (*p* < 0.05) are shown in bold.

**Table 5:** Differences in all outcome measures before and after surgery in the manual canal opening group (data presented as mean ± standard deviation, paired Student’s *t*-test, two-tailed).

	Before	After	*p*	95% Confidence Interval of the Difference	
				Lower	Upper
Leukocytes (×10^9^/L)	10.72 ± 3.07	9.33 ± 2.44	**0.010**	0.36	2.43
Haemoglobin (g/L)	117.23 ± 15.41	103.93 ± 14.06	**<0.001**	9.57	17.03
Platelets (×10^9^/L)	214.47 ± 71.59	211.37 ± 86.87	0.735	−15.48	21.68
D-dimer (µg/L)	11.56 ± 11.12	8.97 ± 8.86	0.304	−2.48	7.68
Prothrombin time (ratio)	0.96 ± 0.13	1.07 ± 0.13	**<0.001**	−0.17	−0.06
aPTT (ratio)	24.10 ± 2.90	24.01 ± 2.66	0.808	−0.61	0.78
Fibrinogen (g/L)	4.19 ± 1.27	5.13 ± 1.20	**0.005**	−1.58	−0.30
Glucose (mmol/L)	7.44 ± 2.54	7.01 ± 2.17	0.489	−0.82	1.68
Urea (mmol/L)	8.01 ± 3.60	8.78 ± 3.40	0.116	−1.75	0.20
Creatinine (µmol/L)	79.50 ± 24.66	93.87 ± 49.18	**0.030**	−27.27	−1.46
eGFR (mL/min/1.73 m^2^)	67.26 ± 21.56	55.57 ± 20.25	**<0.001**	7.12	16.26
NT-proBNP (pg/mL)	385.23 ± 523.33	870.07 ± 750.10	**<0.001**	−644.47	−325.20
C-reactive protein (mg/L)	20.56 ± 23.20	57.07 ± 31.74	**<0.001**	−46.36	−26.65
Sodium (mmol/L)	138.87 ± 3.73	139.43 ± 4.03	0.385	−1.88	0.75
Potassium (mmol/L)	4.06 ± 0.46	4.26 ± 0.54	**0.049**	−0.39	0.00
Chloride (mmol/L)	102.10 ± 4.71	102.83 ± 4.73	0.357	−2.34	0.87
Calcium (mmol/L)	2.27 ± 0.11	2.14 ± 0.11	**<0.001**	0.09	0.17
Phosphate (mmol/L)	1.02 ± 0.18	1.00 ± 0.18	0.727	−0.06	0.09
tP1NP (µg/L)	56.31 ± 22.45	67.38 ± 33.24	**0.022**	−20.40	−1.75
N-mOST (µg/L)	23.68 ± 13.04	26.14 ± 20.73	0.446	−8.99	4.06
β-CTX (ng/mL)	0.64 ± 0.30	0.74 ± 0.28	**0.004**	−0.17	−0.03
BAP (µg/L)	14.42 ± 5.65	17.25 ± 6.41	**<0.001**	−4.05	−1.60

Legend: *p*: significance level; aPTT: Activated partial thromboplastin time; eGFR: estimated glomerular filtration rate; NT proBNP: N-terminal pro-B-type natriuretic peptide; tP1NP: total procollagen type 1 N-terminal propeptide; N-mOST: N-mid osteocalcin; β-CTX: beta-C-terminal telopeptide of type I collagen (B-CrossLaps); BAP: bone-specific alkaline phosphatase. Statistically significant values (*p* < 0.05) are shown in bold.

**Table 6:** Differences in all outcome measures before and after surgery in the mechanical canal opening group (data presented as mean ± standard deviation, paired Student’s *t*-test, two-tailed).

	Before	After	*p*	95% Confidence Interval of the Difference	
				Lower	Upper
Leukocytes (×10^9^/L)	12.53 ± 3.40	13.14 ± 2.93	0.192	−1.55	0.33
Haemoglobin (g/L)	123.57 ± 20.97	99.60 ± 14.72	**<0.001**	19.42	28.51
Platelets (×10^9^/L)	215.07 ± 47.72	201.17 ± 50.43	0.126	−4.13	31.93
D-dimer (µg/L)	13.55 ± 10.81	4.67 ± 3.19	**<0.001**	4.51	13.26
Prothrombin time (ratio)	1.00 ± 0.13	1.11 ± 0.15	**<0.001**	−0.16	−0.06
aPTT (ratio)	23.66 ± 2.54	25.08 ± 3.76	0.065	−2.93	0.09
Fibrinogen (g/L)	3.67 ± 1.03	5.48 ± 1.50	**<0.001**	−2.44	−1.19
Glucose (mmol/L)	7.70 ± 1.88	8.61 ± 2.48	**0.010**	−1.57	−0.23
Urea (mmol/L)	7.53 ± 3.87	10.76 ± 4.10	**<0.001**	−4.31	−2.15
Creatinine (µmol/L)	76.53 ± 22.96	106.67 ± 21.80	**<0.001**	−37.66	−22.61
eGFR (mL/min/1.73 m^2^)	68.90 ± 26.62	57.21 ± 23.71	**<0.001**	8.03	15.36
NT-proBNP (pg/mL)	925.57 ± 1354.64	2204.60 ± 1924.70	**<0.001**	−1804.39	−753.68
C-reactive protein (mg/L)	21.65 ± 27.04	83.66 ± 35.42	**<0.001**	−73.63	−50.39
Sodium (mmol/L)	138.30 ± 5.14	138.63 ± 4.30	0.611	−1.66	0.99
Potassium (mmol/L)	4.34 ± 0.57	4.25 ± 0.41	0.498	−0.18	0.36
Chloride (mmol/L)	101.43 ± 5.42	102.57 ± 4.72	0.096	−2.48	0.21
Calcium (mmol/L)	2.25 ± 0.12	2.10 ± 0.25	**0.001**	0.07	0.24
Phosphate (mmol/L)	0.98 ± 0.25	0.95 ± 0.17	0.578	−0.08	0.14
tP1NP (µg/L)	48.81 ± 17.89	50.97 ± 14.56	0.381	−7.13	2.81
N-mOST (µg/L)	27.15 ± 32.55	20.48 ± 8.21	0.265	−5.34	18.67
β-CTX (ng/mL)	0.61 ± 0.26	0.71 ± 0.26	**0.022**	−0.19	−0.02
BAP (µg/L)	14.69 ± 4.66	20.28 ± 5.75	**<0.001**	−6.79	−4.39

Legend: *p*: significance level; aPTT: Activated partial thromboplastin time; eGFR: estimated glomerular filtration rate; NT proBNP: N-terminal pro-B-type natriuretic peptide; tP1NP: total procollagen type 1 N-terminal propeptide; N-mOST: N-mid osteocalcin; β-CTX: beta-C-terminal telopeptide of type I collagen (B-CrossLaps); BAP: bone-specific alkaline phosphatase. Statistically significant values (*p* < 0.05) are shown in bold.

**Table 7:** Correlations between changes in bone turnover markers and changes in other outcome measures in participants with manual canal opening bone (*n* = 30; Pearson’s correlation test, two-tailed).

	Delta_ tP1NP		Delta_ N-mOST		Delta_ β-CTX		Delta_ BAP	
Delta	r	*p*	r	*p*	r	*p*	r	*p*
Platelets	**0.430**	**0.018**	−0.053	0.782	0.010	0.960	0.201	0.286
D-dimer	−0.208	0.270	**0.570**	**0.002**	−0.018	0.926	−0.044	0.820
Fibrinogen	−0.114	0.548	**−0.387**	**0.034**	0.125	0.510	−0.121	0.524
Urea	−0.335	0.070	−0.086	0.650	**0.417**	**0.022**	0.102	0.592
Creatinine	0.117	0.538	0.003	0.990	**0.446**	**0.014**	−0.016	0.932
eGFR	0.165	0.384	−0.003	0.988	**−0.519**	**0.004**	−0.219	0.246
NT-proBNP	0.170	0.370	−0.021	0.914	0.229	0.224	**0.441**	**0.014**
Calcium	−0.222	0.238	0.063	0.740	0.242	0.198	**0.310**	**0.096**
Phosphate	−0.020	0.916	**−0.393**	**0.032**	**0.374**	**0.042**	−0.011	0.956

Legend: r: Pearson’s correlation coefficient; *p*: significance level; eGFR: estimated glomerular filtration rate; NT proBNP: N-terminal pro-B-type natriuretic peptide; tP1NP: total procollagen type 1 N-terminal propeptide; N-mOST: N-mid osteocalcin; β-CTX: beta-C-terminal telopeptide of type I collagen (B-CrossLaps); BAP: bone-specific alkaline phosphatase. Statistically significant values (*p* < 0.05) are shown in bold.

**Table 8:** Correlations between bone turnover markers and other outcome measures in participants with mechanical canal opening bone (*n* = 30; Pearson’s correlation test, two-tailed).

	Delta_ tP1NP		Delta_ N-mOST		Delta_ β-CTX		Delta_ BAP	
Delta	r	*p*	r	*p*	r	*p*	r	*p*
D-dimer	**0.446**	**0.014**	−0.031	0.870	0.143	0.452	−0.344	0.062
aPTT	**0.484**	**0.006**	−0.206	0.274	0.049	0.796	−0.211	0.264
NT-proBNP	**−0.343**	**0.064**	−0.081	0.670	−0.053	0.780	**0.365**	**0.048**
Calcium	−0.116	0.542	−0.108	0.570	**0.460**	**0.010**	−0.193	0.306

Legend: r: Pearson’s correlation coefficient; *p*: significance level; aPTT: Activated partial thromboplastin time; NT proBNP: N-terminal pro-B-type natriuretic peptide; tP1NP: total procollagen type 1 N-terminal propeptide; N-mOST: N-mid osteocalcin; β-CTX: beta-C-terminal telopeptide of type I collagen (B-CrossLaps); BAP: bone-specific alkaline phosphatase. Statistically significant values (*p* < 0.05) are shown in bold.

## Data Availability

The data presented in this study are available from the corresponding author upon request.
